# Exploring Peripheral Blood Biomarkers for Dementia Diagnosis in Latin American Populations

**DOI:** 10.1002/alz.093462

**Published:** 2025-01-09

**Authors:** Ariel Caviedes, Paulina Orellana, Hernan Hernandez, Andrea Slachevsky, María Isabel Behrens, Diana Matallana, Francisco Lopera, Nilton Custodio, Martin Alejandro Bruno, José Alberto Avila Funes, Leonel Tadao Takada, Stefanie D Pina‐Escudero, Victor Valcour, Kenneth S. Kosik, Bruce L. Miller, Jennifer S. Yokoyama, Agustin Ibañez, Claudia Duran‐Aniotz

**Affiliations:** ^1^ Latin American Institute for Brain Health (BrainLat), Universidad Adolfo Ibañez, Santiago Chile; ^2^ Center for Social and Cognitive Neuroscience (CSCN), School of Psychology, Universidad Adolfo Ibañez, Santiago Chile; ^3^ Latin American Brain Health Institute (BrainLat), Universidad Adolfo Ibañez, Santiago Chile; ^4^ Memory and Neuropsychiatric Clinic (CMYN), Neurology Service, Hospital del Salvador and Faculty of Medicine, Universidad de Chile, Santiago Chile; ^5^ Centro de Investigación Clínica Avanza (CICA), Hospital Clínico Universidad de Chile, Santiago Chile; ^6^ Pontificia Universidad Javeriana, Bogota, Cundinamarca Colombia; ^7^ Neurosciences Group of Antioquia, University of Antioquia, Medellin Colombia; ^8^ Instituto Peruano de Neurociencias, Lima, Lima Peru; ^9^ Instituto de Ciencias Biomédicas (ICBM) Facultad de Ciencias Médicas, Universidad Catoóica de Cuyo, San Juan Argentina; ^10^ Instituto Nacional de Ciencias Médicas y Nutrición "Salvador Zubirán", Mexico, DF Mexico; ^11^ Instituto Nacional de Ciencias Médicas y NUtrición Salvador Zubirán, México, DF Mexico; ^12^ Hospital das Clínicas, University of Sao Paulo Medical School, São Paulo Brazil; ^13^ Memory and Aging Center, UCSF Weill Institute for Neurosciences, University of California, San Francisco, San Francisco, CA USA; ^14^ Memory and Aging Center, Weill Institute for Neurosciences, University of California, San Francisco, San Francisco, CA USA; ^15^ Global Brain Health Institute, University of California, San Francisco, San Francisco, CA USA; ^16^ University of California Santa Barbara, Santa Barbara, CA USA; ^17^ Memory & Aging Center, Department of Neurology, University of California in San Francisco, San Francisco, CA USA; ^18^ Global Brain Health Institute (GBHI), Trinity College Dublin (TCD), Dublin Ireland; ^19^ Latin American Brain Health (BrainLat) Institute, Universidad Adolfo Ibáñez, Peñalolén, Santiago Chile

## Abstract

**Background:**

Dementia, encompassing Alzheimer's disease (AD) and frontotemporal dementia (FTD), poses a substantial public health challenge in Latin America. Barriers such as a shortage of healthcare professionals, limited medical accessibility, and underdiagnosis contribute to the complexity. While biomarkers aligned with the ATN framework (Amyloid, Tau, Neurodegeneration) have revolutionized diagnosis, their cost limits adoption in Latin America. Existing research focuses predominantly on North American or European cohorts, leaving a significant gap for the region. Our groundbreaking study aims to investigate, for the first time, ATN‐related proteins in AD, FTD, and control subjects using blood samples from the ReDLat cohort. This research addresses critical gaps in dementia diagnosis specific to Latin America, guided by insights from the ATN framework.

**Method:**

We enrolled 500 participants (AD, FTD, and healthy controls‐HC) from Argentina, Chile, Colombia, Mexico, and Peru through the Multi‐Partner Consortium to Expand Dementia Research in Latin America (ReDLat). Plasma samples were collected in EDTA tubes, aliquoted, shipped on dry ice, and stored at ‐80°C following standard methods. We assess the plasma levels of Aβ40, Aβ42, p‐tau181, and NfL across groups (AD, FTD, HC) using Lumipulse G600II from Fujirebio, an advanced automated system which detects proteins via chemiluminescence.

**Result:**

Our analysis revealed a significant decrease in the Aβ1‐42/40 ratio between AD and FTD patients compared to HC. Additionally, we observed a significant increase in p‐tau181 levels in both AD and FTD patients relative to HC, with a significant difference between AD and FTD. NfL levels were significantly higher in AD and FTD patients compared to HC.

**Conclusion:**

Our findings demonstrate substantial differences in Aβ1‐42/40 ratio, phosphorylated Tau, and NfL levels between AD, FTD, and control subjects. These distinct biomarker profiles hold promise for distinguishing between various forms of dementia and healthy individuals. Importantly, our study addresses the critical gap in dementia research within LA populations, offering valuable insights into the potential impact of regional factors on biomarker dynamics.

